# Discontinuation of HIV oral pre-exposure prophylaxis: findings from programmatic surveillance within two general population HIV programs in Nigeria

**DOI:** 10.1186/s12889-024-18808-z

**Published:** 2024-05-16

**Authors:** Helen Anyasi, Augustine Idemudia, Titilope Badru, Stanley Onyegbule, Etiemana Isang, Olusola Sanwo, Satish Raj Pandey, Robert Chiegil, Moses Bateganya, Katie Schwartz, Njambi Njuguna, Navindra Persaud, Marya Plotkin, Isa Iyortim, Hadiza Khamofu

**Affiliations:** 1 FHI 360, Abuja, Nigeria; 2 United States Agency for International Development (USAID), Abuja, Nigeria; 3 Centre for Integrated Health Programs (CIHP), Abuja, Nigeria; 4 Excellence Community Education Welfare Scheme (ECEWS), Calabar, Nigeria; 5 Helen Keller International, Asia Pacific Regional Office, Phnom Penh, Cambodia; 6 FHI 360, Washington, DC USA; 7 United States Agency for International Development (USAID), Dar Es Salaam, Tanzania; 8 FHI 360, Nairobi, Kenya

**Keywords:** PrEP, Pre-exposure prophylaxis, HIV prevention, PrEP discontinuation, Sero-discordant relationship, Nigeria

## Abstract

**Background:**

As oral PrEP scales up in Nigeria, information about uptake, use pattern and client preference in a real-world, implementation setting is invaluable to guide refining service provision and incorporation of oral PrEP and other prevention measures into routine health services. To add to this body of knowledge, our study examines factors associated with discontinuation of PrEP among HIV negative individuals across two large scale programs in Nigeria.

**Methods:**

Using program implementation data from two large-scale HIV projects in Akwa Ibom and Cross River states in Nigeria between January 2020 and July 2021, we used logistic regression to explore factors associated with early discontinuation (i.e., stopping PrEP within one month of starting) among HIV-negative individuals who initiated PrEP in the programs.

**Results:**

Of a total of 26,325 clients; 22,034 (84%) discontinued PrEP within the first month. The odds of PrEP discontinuation were higher among clients who enrolled in community-based distribution sites (aOR 2.72; 95% C.I: 2.50–2.96) compared to those who enrolled in program-supported facilities and never married (aOR 1.76; 95% C.I: 1.61–1.92) compared to married clients. Clients who initiated PrEP because of high-risk sexual behaviour (aOR 1.15, 95% C.I 1.03–1.30) or inconsistent use or non-use of condoms (aOR 1.96, 95% C.I 1.60–2.41**)** had greater odds of discontinuing PrEPthan those who initiated PrEP because they were in a serodifferent relationship.

**Conclusion:**

The behavioural and demographic factors associated with early discontinuation of PrEP suggest that risk stratification of pre-initiation and follow up counselling may be helpful in raising continuation rates. On the service delivery side, strategies to strengthen follow-up services provided by community-based distribution sites need to be introduced. Overall, the low continuation rate calls for a review of programmatic approaches in provision of PrEP services in Nigeria.

## Introduction

In 2015, the World Health Organization (WHO) released guidelines recommending Pre-Exposure Prophylaxis (PrEP) as a prevention measure to people at substantial risk of HIV infection [1]. Since then, the global strategy to reach “zero new infections” has included combination prevention which entails the use of behavioural, biomedical, and structural methods [2, 3]. Oral PrEP is recommended for key populations, such as men who have sex with men and sex workers; it is also recommended as an means to decrease the chances of infections amongst heterosexual sero-different couples [4]. Since 2015, a growing number of countries have adopted WHO recommendations on PrEP into national guidelines, and oral PrEP has become available through pilot projects, implementation studies, and national programmes [5] and over 5.6 million persons have initiated PrEP since then [6]. In Nigeria, oral PrEP was included in the National HIV/AIDs Prevention, Care, and Treatment guidelines as a biomedical prevention method in 2020 [7] and since then over 380,000 people in Nigeria have initiated PrEP [8].

As oral PrEP scales across countries, more information about acceptability, uptake, use pattern and client preferences has emerged from pilots, demonstration studies and national programmes. These findings are useful for refining service provision and providing learnings on how oral PrEP and other prevention products or methods can be effectively incorporated into routine health services. In 2018, across early demonstration projects in seven countries, there was a high continuation rate: in some cases, up to 94% of individuals in a sero-different relationship were still using PrEP at 3 months [9]. However, more recently, notable and consistent findings across many studies and programmes indicate that PrEP users often discontinue oral PrEP within 1 to 3 months, and continuation rates are low [10–13]. Researchers have investigated client-, provider- and system-level perspectives to understand factors that affect oral PrEP continuation; studies done in Eswatini, Kenya, South Africa, US and Uganda highlight reasons such as stigma-related barriers to accessing PrEP at ART clinics, issues around inconvenient clinic locations or operating hours, long wait times, and low numbers of prescription leading to need for frequent refills [14–16]. Client-level factors include: changes to self-perceived HIV risk, side effects [17], daily pill burden [10, 15], concealing pills from partner or partner’s insistence to discontinue PrEP [18, 19]. As similar findings emerge, HIV prevention experts call for PrEP use to be viewed as less continuous, with considerations for variable risk and prevention method switching [18]. Current indicators for oral PrEP measure the number of individuals newly initiated on PrEP, seroconversion rates and PrEP continuation rates [19] – these indicators largely do not account for discontinuous use patterns and effectiveness during periods of use.

To improve oral PrEP service delivery and draw lessons for introduction of new near-market HIV prevention products, there are calls for learnings that reveal opportunities, close program design gaps and unearth challenges in PrEP provision, especially in real-world settings [20]. Our study, which examined factors associated with discontinuation of PrEP across two large-scale programs in Nigeria, used a programmatic surveillance approach in which routine program data were analyzed to find causes associated with discontinuation. The findings represent real-world implementation which can be used to improve program design and support policy makers to anticipate needs of future PrEP programs.

## Methods

### Study design

Surveillance is a descriptive study method, with an emphasis on feedback based on detection of cases, where a population is monitored for a for a condition to inform and plan for prevention or control activities [21]. The study design used for the current study can best be described as programmatic surveillance, an approach which has been described in other studies [22]. In this approach, surveillance is applied to programmatic data to identify, feedback, and improve programs based on findings. Surveillance can be either active (often employed during outbreaks or emergencies) or passive (with a more descriptive orientation). This study employed a passive surveillance approach to programmatic data, as cases of PrEP discontinuation were not actively sought and contacted in real time. Instead, this analysis identified discontinuation of PrEP users among eligible, HIV-negative people reached through two large-scale HIV projects, with the intention of improving program approaches. The programmatic data used in the study were collected from January 2020 to July 2021, in Akwa-Ibom and Cross River States of Nigeria.

### Setting

Akwa Ibom and Cross River States have HIV prevalence that is higher than the national average (national prevalence 1.4%, Akwa Ibom and Cross River states 5.5% and 2.2%, respectively) [23]. Based on prevalence and the states’ epidemic dynamics, the United States Agency for International Development (USAID) through the Strengthening Integrated Delivery of HIV/AIDS Services (SIDHAS) and Meeting Targets and Maintaining Epidemic control (EpiC) projects, launched a HIV “surge” implementation in Akwa Ibom and Cross River states [24, 25]. The surge implementation included both facilities (tertiary, secondary, private-for-profit, and primary health facilities) as well as massive community case finding and treatment saturation in line with epidemic drivers. Community-based service delivery was done through two models: community pharmacies, and community ART management (CAM) teams [26]. Community pharmacies were static structures already existing in the communities, while CAM teams were mobile, roving sites linked to health facilities in a hub-and-spoke model for supplies and reporting. Using this model, comprehensive HIV services, with clinicians providing clinical supervision, were provided within communities to clients who were unable or unwilling to go to health facilities.

In 2020, within the context of the ART surge, oral PrEP was introduced in these states. PrEP was offered through the same community-based and health facility delivery models that provided HIV testing and treatment services; oral PrEP was offered to partners of index clients and high HIV-risk individuals identified at both facility service delivery points and community-based HIV testing services. To further clarify the programme population, index clients are individuals diagnosed as HIV positive, aware of their status, enrolled in the HIV treatment program, and shared contacts of partners for notification about HIV risk. High HIV-risk individuals are persons identified during HIV counselling and testing as engaging in unprotected/condomless penetrative sex (vaginal, anal, or oral), having multiple sexual partners, engaging in transactional sex and/or has been treated for sexually transmitted infection in the last 6 months.

### PrEP service provision and implementation

The program prioritized HIV-negative partners of index clients and other individuals identified as “at-high-risk” for HIV for oral PrEP services at both health facilities and within supported communities. This analysis includes all individuals who initiated PrEP between January 2020 and July 2021. The majority of these were identified during index case counselling and partner testing where service providers engage and counsel serodifferent^1^ sexual partners on risk reduction. Others included individuals who received HIV risk reduction counselling and testing at either the health facility or within the community and upon receiving a negative HIV test decided to use PrEP.

### Patient monitoring and follow-up

On enrollment, the PrEP users described in this study were provided with one month’s worth of pills and asked to return one-month post-initiation for a follow-up visit. Individuals were asked to reach out to the health facility or community-based team in the event of an adverse drug reaction. Providers counseled on risk reduction for HIV and other sexually transmitted infections, advocated for condom use along with PrEP, conducted HIV testing and screening for renal function. Providers halted PrEP to clients who seroconverted, had severe adverse effects, or failed renal function screening. At these visits, the client was free to decline or continue PrEP use. If PrEP use was declined, no further follow-up visits were scheduled, and the individual was informed that they are free to re-start at any time. If the client wished to continue, a three-month refill was given. All subsequent visits were quarterly if the client continued to return for refills. In the electronic medical records, PrEP users’ follow-up status was categorized as either “ongoing use” or “discontinued PrEP”. A person was said to have discontinued PrEP when the individual declined further refill of PrEP or did not return for their follow-up appointment.

### Data collection and management

Before analysis, de-identified data for the cohort of persons who had started PrEP between January 2020 and July 2021 were extracted from the Lafiya Management Information System (LAMIS), one of the national electronic medical records (EMR) systems. The LAMIS data contained client’s socio-demographics, status of taking PrEP, as well as indications for taking PrEP. As part of internal quality control measures, data were cleaned weekly to ensure optimum data quality, this entailed but is not limited to the mop-up of missing data.

### Study outcomes

The primary outcome measure was early discontinuation of PrEP by the one-month follow up visit post initiation. In this study, early discontinuation was defined as clients who did not return for a refill visit one month after initiating PrEP.

### Data analysis

Descriptive statistics such as proportion, median, and interquartile range were used to summarize participants’ characteristics. Pearson’s chi-square test was used to examine associations between socio-demographic characteristics and PrEP discontinuation. We used multivariable logistic regression to investigate associations between independent variables and PrEP discontinuation. Bivariate analysis was conducted on independent variables and those significantly associated with PrEP discontinuation at *p *< 0.10 were entered into multivariable model. However, important variables of known clinical importance were included if *p* > 0.10. Independent variables considered were age, sex, educational status, occupation, marital status, initiation setting and indications for initiating PrEP. All analyses were stratified by gender to explore whether there are gender differences affecting PrEP discontinuation. Variables with *p*-value < 0.05 were considered as associated factors.

### Ethics

This analysis of routine program data was given a non-research determination FHI 360 Office of International Research Ethics (OIRE). Informed consent was not required as no people were interviewed for this study – only aggregate data were used in the analysis. Authors had no access to identifiable data of clients included in this study.

## Results

From January 2020 to July 2021, 26,325 HIV-negative individuals in program-supported facilities and community-based distribution sites started PrEP. Of these, more than half (54%; 14,099) were enrolled in program-supported health facilities, while 46% (12,226) were enrolled at community-based distribution sites. Median age was 31 years (IQR: 27–38), 61% (16,111) were males, 54% (14,300) never married, 81% (19,376) had at least secondary education, and 55% (13,235) were unemployed (Table 1).

**Table 1:** Socio-demographic characteristics of individuals initiated on PrEP in Akwa Ibom and Cross River States of Nigeria (January 2020 – July 2021)

	**Female (*N* = 10,214)** **Number (%)**	**Male (*N* = 16,111)** **Number (%)**	**Total (*N* = 26,325)** **Number (%)**
**State**			
Akwa Ibom	8,347 (81.7)	12,825 (79.6)	21,172 (80.4)
Cross River	1,867 (18.3)	3,286 (20.4)	5,153 (19.6)
**PrEP Initiation setting**			
Health Facilities	5,610 (54.9)	8,489 (52.7)	14,099 (53.6)
Community-based distribution sites	4,604 (45.1)	7,622 (47.3)	12,226 (46.4)
**Age category (years)**			
15–24	2,165 (21.2)	1,552 (9.6)	3,717 (14.1)
25–29	2,986 (29.2)	3,286 (20.4)	6,272 (23.8)
30–34	2,325 (22.8)	3,819 (23.7)	6,144 (23.3)
35–39	1,478 (14.5)	2,969 (18.4)	4,447 (16.9)
≥ 40	1,260 (12.3)	4,485 (27.8)	5,745 (21.8)
Median age (IQR)	29 (25–35)	33 (28–40)	31 (27–38)
**Education attained**^a^ **(*N* = 23,979)**			
None	470 (5.1)	804 (5.5)	1,274 (5.3)
Primary	1,309 (14.1)	2,020 (13.7)	3,329 (13.9)
Secondary	6,340 (68.3)	9,905 (67.4)	16,245 (67.8)
Post-secondary	1,161 (12.5)	1,970 (13.4)	3,131 (13.1)
**Marital status**			
Never married	5,854 (57.3)	8,446 (52.4)	14,300 (54.3)
Currently married	4,261 (41.7)	7,538 (46.8)	11,799 (44.8)
Previously married	99 (1.0)	127 (0.8)	226 (0.9)
**Employment status**^a^ **(*N* = 24,095)**			
Employed	3,158 (33.9)	5,801 (39.2)	8,959 (37.2)
Unemployed	5,212 (56.0)	8,023 (54.3)	13,235 (54.9)
Student	942 (10.1)	959 (6.5)	1,901 (7.9)

^a^Complete data analyzed

Providers conducted an individualized risk assessment to ascertain the indication for initiating PrEP. Majority (61%) initiated PrEP because they were in a serodifferent relationship.

**Fig. 1:**
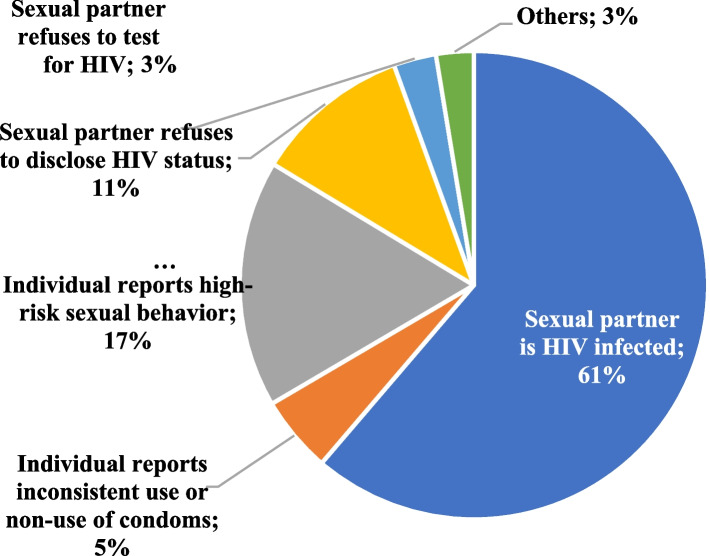
Reasons for initiating PrEP in Akwa Ibom and Cross River States in Nigeria (January 2020 – July 2021)

Other reasons reported for starting PrEP were partner non-disclosure^2^ (11%), high risk sexual behaviour (17%), partner unwilling to undergo HIV testing (3%), inconsistent or non-use of condoms (5%) (Fig. 1).

### PrEP discontinuation within one month

Approximately 84% (22,034) of those initiated on PrEP discontinued within one month. In the adjusted analysis, the odds of discontinuing PrEP were higher among those who enrolled in community-based distribution sites compared to those who enrolled in program-supported facilities [ 91.3% (11,161) vs. 77.1% (10,873), *p* < 0.001; adjusted odds ratio (aOR) 2.72; 95% C.I: 2.50–2.96]. For demographic factors, never married clients had greater odds of discontinuing PrEP compared to married clients [88% (12,583) vs. 78.5% (9,262), *p* < 0.001; adjusted odds ratio (aOR) 1.76; 95% C.I: (1.61–1.92)]. In terms of behavioral factors, early discontinuation of PrEP was highest among those whose indication for starting PrEP was inconsistent use or non-use of condoms (91.7%, 1,278), followed by high-risk sexual behaviour (90.4%, 4,067) compared to other reasons (*p* < 0.001). Odds of discontinuing PrEP was greater for clients who initiated PrEP because of high-risk sexual behaviour (aOR 1.15, 95% C.I 1.03–1.30) or inconsistent use or non-use of condoms (aOR 1.96, 95% C.I 1.60 -2.41) than those who initiated PrEP because they were in a serodifferent relationship (Table 2). Female-specific correlates of PrEP discontinuation included marital status (aOR 1.87, 95% C.I 1.62–2.14), enrolment setting (aOR 2.83, 95% C.I 2.46–3.24), and indication for PrEP initiation (aOR 2.06, 95% C.I 1.47–2.88). Among male, same factors were associated with PrEP discontinuation; marital status (aOR 1.70, 95% C.I 1.52–1.90), enrolment setting (aOR 2.65, 95% C.I 2.39–2.94), and indication for PrEP initiation (aOR 1.90, 95% C.I 1.46–2.46).

**Table 2:** Factors associated with early discontinuation of PrEP among Users in Akwa Ibom and Cross River States of Nigeria (January 2020 – July 2021)

Characteristic	Discontinued PrEP within one month- n (%)	Overall			Female			Male		
		**OR (95% C.I)**	**AOR (95% C.I)**	***P*-value**	**OR (95% C.I)**	**AOR (95% C.I)**	***P*-value**	**OR (95% C.I)**	**AOR (95% C.I)**	***P*-value**
**State**										
Akwa Ibom	17,819 (84.2)	1	1		1	1		1	1	
Cross River	4,215 (81.8)	0.85 (0.78–0.92)	**0.68 (0.62–0.75)**	**< 0.001**	0. 73 (0.64–0.83)	**0.61(0.52–0.71)**	**< 0.001**	0.92 (0.83–1.02)	**0.73 (0.65–0.83)**	**< 0.001**
**Setting**										
Health Facilities	10,872 (77.1)	1	1		1	1		1	1	
Community based distribution sites	11,161 (91.3)	0.85(0.78–0.92)	**2.72 (2.50–2.95)**	**< 0.001**	3.29 (2.91–3.72)	**2.83(2.46–3.24)**	**< 0.001**	3.01 (2.74–3.31)	**2.65 (2.39–2.94)**	**< 0.001**
**Gender**										
Female	8,564 (83.9)	1	1							
Male	13,470 (83.6)	0.98 (0.92–1.05)	0.96 (0.89–1.04)	0.37						
Age, mean/SD (years)	32.8 (8.5)	0.98 (0.97–0.98)	0.10 (0.99–1.00)	0.75	0.98 (0.97–0.99)	1.01 (0.99–1.02)	0.10	0.98 (0.97–0.98)	0.99 (0.98–1.00)	0.13
**Education attained**										
None	1,059 (83.1)	1	1		1	1		1	1	
Primary	2,784 (83.6)	1.04 (0.87–1.23)	1.01 (0.83–1.21)		1.02 (0.76–1.37)	0.92 (0.68–1.26)		1.22 (0.98–1.52)	1.05 (0.83–1.33)	
Secondary	13,933 (85.8)	1.22 (1.05–1.43)	0.95 (0.79–1.14)	0.52	1.06 (0.79–1.40)	1.05 (0.78 -1.42)	0.40	1.02 (0.82–1.27)	0.89 (0.71–1.12)	0.30
Tertiary	2,658 (84.9)	1.14 (0.96–1.36)	1.03 (0.88–1.21)		1.22 (0.95–1.58)	1.08 (0.83 -1.41)		1.22 (1.01–1.48)	0.99 (0.81–1.22)	
**Marital status**										
Currently married	9,262 (78.5)	1	1		1	1		1	1	
Never married	12,583 (88.0)	2.01 (1.88–2.15)	**1.76 (1.61–1.92)**	**< 0.001**	2.11 (1.89–2.35)	**1.87 (1.62–2.14)**	**< 0.001**	1.95 (1.79–2.12)	**1.70 (1.52–1.90)**	**< 0.001**
Previously married	189 (83.6)	1.40 (0.98–1.99)	1.03 (0.71–1.48)		1.37 (0.81–2.31)	0.95 (0.54–1.66)		1.44 (0.89–2.33)	1.09 (0.66 – 1.79)	
**Occupation**										
Unemployed	10,993 (83.1)	1	1	**< 0.001**	1	1		1	1	
Employed	7,785 (86.9)	1.35 (1.25–1.46)	**1.48 (1.37–1.61)**		1.42 (1.25–1.61)	**1.51 (1.32–1.73)**	** < 0001**	1.32 (1.20–1.45)	**1.47 (1.32–1.62)**	**< 0.001**
Student	1,724 (90.7)	1.99 (1.69–2.33)	**1.60 (1.35–1.91)**		2.08 (1.64–2.63)	**1.79 (1.38–2.32)**		1.90 (1.52–2.37)	**1.48 (1.17–1.88)**	
**Indication for PrEP**										
Serodifferent relationship	13,424 (83.2)	1	1		1	1		1	1	
Inconsistent use or non-use of condom	1,278 (91.7)	2.24 (1.84–2.72)	**1.96 (1.60–2.41)**		2.29 (1.68–3.13)	**2.06 (1.47–2.88)**		2.20 (1.71–2.83)	**1.90 (1.46–2.46)**	
Reports high-risk sexual behaviour	4,067 (90.4)	1.89 (1.70–2.11)	**1.15 (1.03–1.30)**	**< 0.001**	2.16 (1.81–2.60)	**1.29 (1.06–1.58)**		1.75 (1.53–2.00)	1.08 (0.93–1.25)	
Partner non-disclosure	2,024 (71.1)	0.50 (0.45–0.54)	0.95 (0.76–1.18)		0.71 (0.53–0.95)	0.80 (0.58–1.09)	**< 0.001**	0.98 (0.73–1.30)	1.11 (0.82–1.51)	**< 0.001**
Partner unwilling to undergo HIV testing	693 (89.5)	1.72 (1.36–2.18)	0.97 (0.85–1.12)		0.52 (0.45–0.60)	1.14 (0.90–1.43)		0.48 (0.43–0.54)	0.88 (0.73–1.05)	
Others	508 (74.3)	0.84 (0.69–1.03)	1.09 (0.85–1.40)		2.18 (1.48–3.21)	1.38 (0.92–2.07)		1.47 (1.09–1.97)	0.92 (0.67–1.26)	

Independent variables including age, educational status, occupation, marital status, initiation setting and indications for initiating PrEP were adjusted for

## Discussion

The purpose of this work was to examine factors associated with discontinuation of oral PrEP across two large scale HIV programs in Nigeria; these findings illustrate real-world programmatic implementation in these two states of Nigeria. Over 80% of persons initiated on oral PrEP discontinued within the first month of commencement. Study findings in US, Kenya and South and other meta-analyses of studies have also revealed high drop-off rates PrEP within 1 to 3 months [10–13]. Early discontinuation was associated with a system-based factor: starting PrEP through community distributors rather than at a health facility; as well as demographic or behavioural factors: being unmarried, engaging in high-risk sexual behaviour, inconsistent or non-use of condoms.

While both programs delivered oral PrEP through health facilities and in community-based settings, there were slightly more persons who initiated oral PrEP in health facility settings. However, the number of people accessing PrEP through community service delivery points (over 12,000 in under two years) does demonstrate that oral PrEP can be initiated within community service delivery points. Indeed, oral PrEP HIV prevention programs in South Africa [27], Cameroon [28] and Kenya [29] have recorded successes in generating demand, increasing awareness, and improving access to PrEP through community models. This study also revealed, however, that persons who initiated oral PrEP in the community-based settings had a higher rate of discontinuation. Differentiated service delivery models for HIV provide client-centric service delivery and decentralizes access to HIV care and treatment especially in special circumstances such as hard to reach terrains [25] and during the COVID-19 pandemic [30]. This study, however, provides an opportunity to probe the strength of these novel channels of delivery to meet the unique prevention needs associated with PrEP. Needs associated with PrEP continuation are very different from those of PLHIV on lifelong ART. This study highlights the importance of understanding the circumstances impact continuation, making follow up and discontinuation strategies for community settings important.

Study findings showed that the absence of a formal relationship status was an influencing factor in PrEP discontinuation: the highest number of individuals who stopped PrEP were “never married,” and “never married” persons were almost two times more likely to discontinue PrEP within one month of initiation compared to married users. However, it is important to note that the only classifications provided for relationship status in both projects’ medical records are: “married,”, “previously married” or “never married.” This greatly limits the range of options that exist in sexual relationship statuses, for instance, there are co-habiting sexual partners who are not married. Accounting for informal sexual relationships is important when designing programs, developing counselling materials, training service providers and documenting service provision. Studies in both the US and Tanzania note that lack of a formal relationship status, age, and sex [31], especially being young and female [32] raises issues around provider biases related to social mores in the provision of PrEP. Therefore, broadening definitions for sexual relationship status in counselling and providing prevention services could be helpful to address issues around stigma in service provision and remove barriers in access and service delivery especially for single, unmarried young women.

Lastly, it is important to point out that though near perfect adherence on ART is important to achieve HIV viral suppression, ‘imperfect’ use of PrEP has been found to have a significant impact on reducing new infections at the population-level [13, 33, 34]. This has caused researchers and prevention experts to tilt towards adherence at times of risk; this is known as Prevention-Effective adherence [34, 35]. A PrEP user can be empowered with adequate information for adherence during periods of risk exposure when PrEP is most required [35]. Counselling at both health facility and within the community-based settings at initiation should integrate continued willingness to use PrEP and the development of strategies for when PrEP use is most likely to be discontinued with the possibility for re-start if risk is re-introduced. Discontinuations can therefore be seen as the end of a successful short-term intervention rather than an interruption [36].

### Strengths and limitations

This study provided findings from programmatic surveillance: findings on PrEP continuation were used to both systematically assess and to feed back into program improvements. The results provide insight into real-world implementation and provide a realistic look at PrEP use in these settings in Nigeria. The scale of the programs allowed for a large population for which usage patterns could be analysed. Because the data collected for these studies were individual level data, analysis based on a variety of characteristics were possible. The limitation is related to the design of programmatic surveillance. All findings are based on available programmatic data that has a limited number of variables. The primary purpose of the program was to provide services, so data collected, while verified, may have lacked some of the rigor of a study with a primary intention of research. The study was not designed to be representative of other populations, groups, or settings in Nigeria. Despite this limitation, this study provides important information that can be used to improve PrEP continuation rates in these settings and settings which are similar to these two states.

## Conclusions

Early discontinuation of PrEP was high, especially for individuals who commenced in the community, and never married. It was also high among individuals who reported inconsistent use or non-use of condoms. It is recommended that PrEP services should be strengthened with robust counselling around use patterns and perceived risk at initiation and at every subsequent follow-up visit. PrEP discontinuation can be seen as the successful endpoint of intervention if preceded by adequate counselling for risk determination. We also recommend broadening definitions and language for sexual relationship status other than marital status in counselling and prevention service provision to shape provider communication and support for individuals needing and using PrEP services especially for single, unmarried young women. Further exploration on user perspectives around reasons for discontinuation and perceived HIV risk is important to generate useful insights into preferences and behaviours around prevention. We conclude that it is important to explore measures that account for non-continuous use of PrEP, depending upon use pattern and associated risk.

## Data Availability

The data that were used for this analysis were extracted from the Lafiya Management Information System (LAMIS) which is owned by the Federal Ministry of Health, Nigeria. The subset of data that was used for this analysis will be made available upon request by emailing Helen Anyasi (hanyasi@fhi360.org).

## Footnotes

1 their sexual partner was HIV infected and they were not.

2 Partner non-disclosure refers to a situation where an HIV-positive individuals fails to tell their sexual partner(s) about the HIV-positive sero-status.
